# Augmented Reality for Enhanced Visualization of MOF Adsorbents

**DOI:** 10.1021/acs.jcim.3c01190

**Published:** 2023-09-26

**Authors:** Lawson T. Glasby, Rama Oktavian, Kewei Zhu, Joan L. Cordiner, Jason C. Cole, Peyman Z. Moghadam

**Affiliations:** † Department of Chemical and Biological Engineering, The University of Sheffield, Sheffield, S1 3JD, United Kingdom; ‡ Department of Chemical Engineering, University College London, London, WC1E 7JE, United Kingdom; § Cambridge Crystallographic Data Centre, Cambridge, CB2 1EZ, United Kingdom

## Abstract

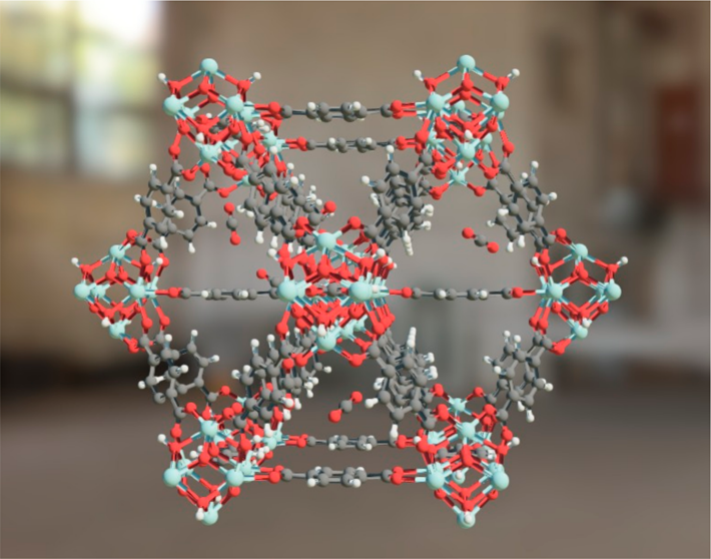

Augmented reality (AR) is an emerging technique used to improve visualization and comprehension of complex 3D materials. This approach has been applied not only in the field of chemistry but also in real estate, physics, mechanical engineering, and many other areas. Here, we demonstrate the workflow for an app-free AR technique for visualization of metal–organic frameworks (MOFs) and other porous materials to investigate their crystal structures, topology, and gas adsorption sites. We think this workflow will serve as an additional tool for computational and experimental scientists working in the field for both research and educational purposes.

## 1 Introduction

Porous materials such as metal–organic frameworks (MOFs), covalent organic frameworks (COFs), zeolites, silicates, polymers, and aerogels are characterized by their pore space and functionality. These properties make them desirable for a broad range of applications within the fields of chemistry, materials science, and engineering. One particularly popular subclass, MOFs, are highly ordered porous materials comprised of metal ions or clusters connected by organic ligands. MOFs have received considerable interest over the past 25 years due to their structural diversity, high surface area, and tunable properties making them suitable materials for a broad range of adsorption applications including gas storage,^1−3^ separation,^4−6^ sensing,^7−9^ and catalysis.^10,11^ As of April 2023, the Cambridge Structural Database (CSD) has seen the addition of over 27,000 3D experimentally synthesized MOFs and, due to the porous nature of these structures, many are studied as potential candidates for gas adsorption and separation applications.^12^

Details regarding MOFs’ structural network, pores, surface chemistry, and adsorption sites are critical pieces of information when investigating the adsorption properties of these structurally complex materials, and this information is often used in conjunction with simulation software to predict gas adsorption properties.^13−15^ Adsorption simulation snapshots can be used to analyze energetically favorable adsorption sites in porous materials, and AR can create aesthetic representations of pores along with the adsorbed molecules. AR enhances spatial understanding by enabling visualization and manipulation of complex structures under specific conditions in 3D, offering additional insights when studying structure–property relationships and guest–host interactions. The use of AR has been previously reported for molecular structures using an app,^16^ for polymers using an app-free technique,^17^ as well as published workflows that build VR models which can also be viewed in AR:^18^ these are typically used for educational purposes as a teaching medium.^19−21^ Here, following the work of Roshandel et al.,^17^ we developed a protocol for app-free AR models that can be used to display MOFs under adsorption conditions or represent crystal structures in conjunction with their topology which can be viewed using a smartphone running Android or iOS.

**Figure 1:**
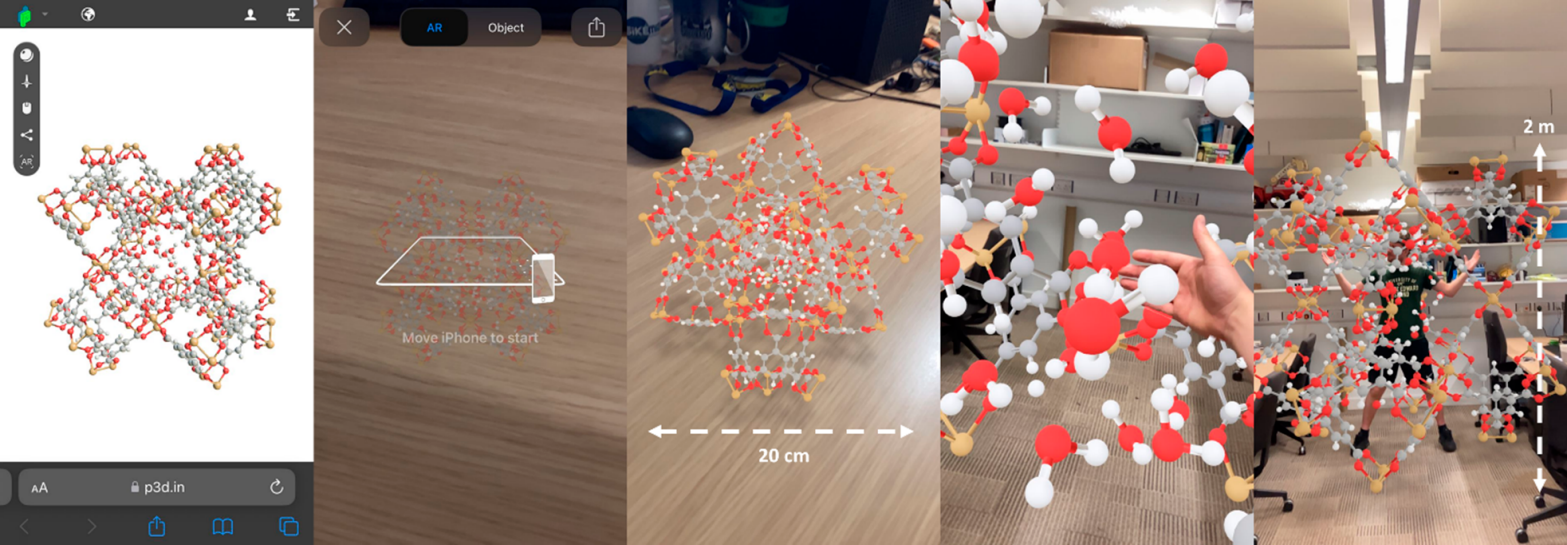
After scanning the QR code using a smart phone, Cu-BTC is projected in augmented reality (AR), demonstrated on the table (20 cm diameter) and in much larger scale in an office with a student standing inside the pores (2 m diameter). Here, the pores contain water molecules where the structure was simulated for water adsorption.

Before we discuss the workflow for creating AR models of MOFs, let us demonstrate an example for AR gas adsorption visualization in an educational setting. Figure 1 shows the application of AR for water adsorption visualization in a prototypical MOF called Cu-BTC (Cu and benzene-1,3,5-tricarboxylate (BTC)), from the point of scanning the QR code to scaling the structure to fill the room. The modeling software enables the user to view the crystal structure online in 3D, or to project AR into the room either using small dimensions as shown on a desk or using large dimensions such that a person can easily fit inside the pores. Another great immersive feature is the shadowing and depth perception capabilities within the AR platform that make it an ideal tool for use in education, enabling the demonstration of complex structures in a classroom or lecture setting.

## 2 Modeling MOFs for Use in AR

This paper explains how to create simple but aesthetic AR representations of MOFs and other crystalline materials, without the need for any significant coding knowledge. Most of the tools we use are freely available, and where licensed software packages are used, freeware alternatives can be obtained. While the workflow in this project consisted primarily of obtaining CIF or PDB files from the CSD 3D MOF subset,^22^ it is possible to create these AR models using any CIF or PDB file from other sources. A detailed guide showing step-by-step instructions on how to obtain and process these files can be found in the Supporting Information (SI), followed by further explanations regarding the conversion from these chemical data files into file formats that can be used for the visualization stages, the generation of quick response (QR) codes, and the subsequent hosting of the AR maps. From the initial stage of obtaining the desired structure from the CSD, to distribution of a QR code directing your audience to the online AR resource, creating these representations can take less than 1 h per structure.

### 2.1 Crystal Structure Modifications

The initial stage of AR visualization begins by selecting a MOF, opening the corresponding CIF/PDB file, and expanding the representation to its unit cell or supercell. In this process, we select structures from CSD ConQuest and use Mercury^23^ as a key tool to implement corrections on the crystal structures (e.g., addition of missing hydrogens or removal of bound/unbound solvents, if required), followed by the repair of broken or unusual chemical bonds. Often, PDB outputs have certain configured bonding patterns, and the CSD software suite is ideal for fast and easy corrections to these abnormalities. It is essential that all models are corrected in this preliminary stage so that errors are not carried forward into the AR representations, and it is recommended to manually check all atoms in the structure even after autocorrection to ensure there are no “floating” atoms remaining or undesired solvents still present in the pores. It is also possible to “snip-off” any over branching linkers that exceed the dimensions of the unit cell at this stage to ensure uniformity of the crystal structure.

### 2.2 Conversion Processes from CIF/PDB to FBX

Once all bonding information has been determined and corrected, and the structure representation has been chosen (e.g., ball and stick, ellipsoidal, wireframe, etc.) it is possible to export the file as either a PDB or CIF. Once the file is saved as a PDB or CIF file, it can be opened in Jmol,^24^ an open-source file conversion and visualization software. From Jmol, detailed instructions in the SI explain how to save and export this file as an object (OBJ) file, but note that after this point, it becomes much more difficult to make any chemical modifications to the structure: bonding can still be corrected in Jmol as detailed in the software documentation. The OBJ file can then be imported into the 3D modeling freeware, Blender.^25^ (An alternative method which, requires the installation of the Atomic Blender plugin, can skip the Jmol step as it is possible to directly import PDB files into Blender: through various trials we determined that the OBJ technique produces equally, if not better, representations in the final stages.) Once the OBJ file is added to Blender, the remaining stages involve the removal of the light and camera layers, followed by a check of structure face count. If the face count exceeds 750,000, the image will not render correctly into AR so the “decimate” function can be used to reduce the number of faces. For best results, keeping the number of faces as close as possible to 750,000 is recommended. The reduced file can then be manipulated so that the orientation is presented as desired, before it is exported as an FBX file.

### 2.3 Publication on p3d.in and QR Code Generation

**Figure 2:**
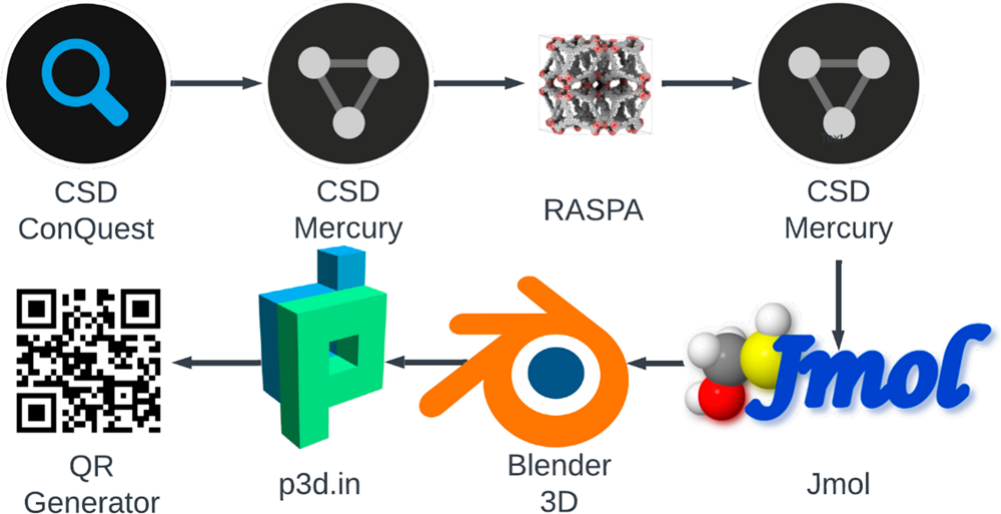
Graphical representation of the AR visualization workflow for MOF adsorbents. We begin from initial structure selection in CSD ConQuest followed by exportation of the unit cell for use in RASPA via Mercury, structure cleanup, file format conversions in Jmol, modeling and export in Blender, and finally upload to the p3d.in platform and generation of a custom QR code.

The publication stage is very simple but requires a free (or paid) subscription to p3d.in, an online 3D model hosting platform with built-in AR functionality.^26^ The FBX file can be uploaded, and the structure’s final orientation and color scheme can be selected. It is also possible to customize the online viewer to display the structure with different background colors, select mouse/keyboard configurations for controllability, and determine the scale of AR representation. Each file is then given a unique URL which can be assigned a static QR code using any, freely available QR code generator (we used https://www.the-qrcode-generator.com/) to create a QR link to the AR enabled structure. Figure 2 shows a graphical summary of the workflow we developed to create complex AR representations of MOFs and their gas adsorption snapshots using the molecular simulations software package, RASPA.^27^

## 3 Applications of AR in Representing MOFs

AR has seen increasing use in the field of 3D chemical and molecular structure modeling in recent years, originating from a very limited number of publications to an increasingly popular and more widespread audience.^21^ We previously introduced a step-by-step guide to use AR in the field of MOFs in 2022;^28^ however, this process was tedious and complex requiring the development and publication of an app. The current approach follows an easier, app-free, workflow. Although it would be straightforward to produce AR representations for basic MOF structures, it is possible to make these tools more useful. For example, the representation of molecular interactions between gases adsorbed in MOFs are mostly represented in 2D which can make it difficult to fully appreciate the complex interplay between structural network, pore shapes and sizes, surface chemistry, and preferential adsorption sites. Clearly, a 3D AR technology to assist in the detection of adsorption sites or topological features of MOFs provides better understanding of the adsorption phenomena and the pore environment.

### 3.1 Crystal Representation

For this article, we created several interesting and diverse AR representations of MOFs under various conditions to demonstrate the relevance and reach of AR in the field, from basic MOF visualizations to more complex representations of their topologies and gas adsorption. Table 1 contains a selection of materials created using a combination of structure preparation approaches, demonstrating the range of uses for AR visualization. The initial representations are taken directly from the CSD 3D MOF subset with only minor modifications. Rows 1–3 in Table 1 show how AR can be used to visualize MOF crystal structures (e.g., ZIF-8, MIL-54, and CPO-27) without the need for complex modifications to CIF files from the CSD. The choice of crystal structures represented here took several factors into consideration. For better aesthetics and clarity, we avoided materials with large unit cells because of computational expense—when converting large unit cell structures to OBJ files, the file size can exceed several hundred MBs which makes rendering more difficult. The structures shown in Table 1 are illustrative examples, we mainly picked materials that are well-known in the MOF community for gas adsorption applications with CIFs that are easily accessible.

**Table 1:**
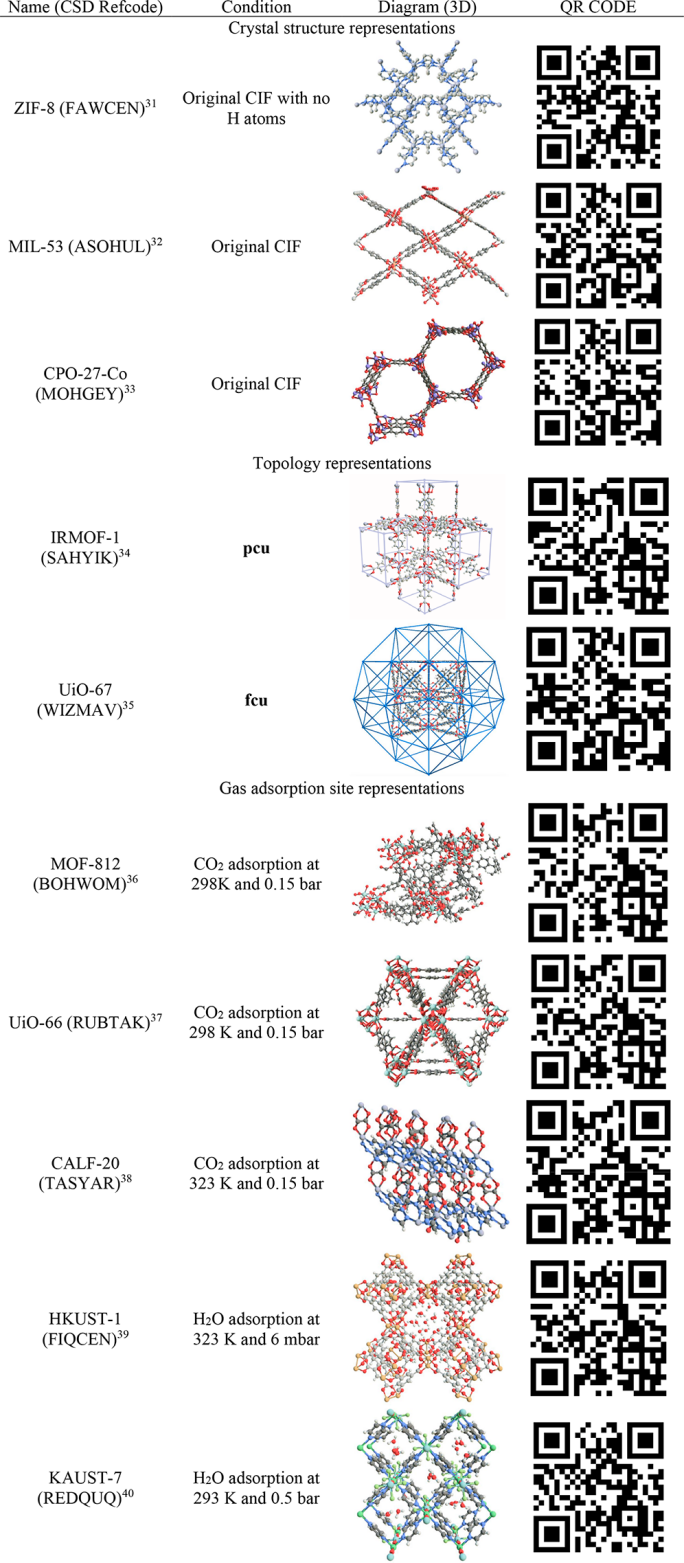
Selection of MOFs Visualized in AR under Different Conditions^a^

a In original form taken directly from the CSD, with topological representations intertwined with the original structures, and for gas adsorption applications.^31−40^

### 3.2 Topology Representation

Another interesting use of AR is for the comparison between the complex crystal structure of MOFs and their underlying topologies. The creation of “MOF plus topology” AR representations involves the use of software such as ToposPro^29^ or CrystalNets^30^ to generate the underlying net for a given CIF. It is essential that topological nets correctly match the dimensions of the CIF so they can be overlaid together for AR representation. Once the topology is determined, the next stage is to convert both the CIF and its corresponding net into separate OBJ files where they can then be layered together in Blender for export into a single FBX file. Topology nets are typically created as CGD or MOL2 files that can be imported into CSD Mercury, before being exported as PDB files for use in Jmol. One disadvantage of this method is that the underlying net representation cannot later be switched on/off in the free version of p3d.in, but it remains an interesting tool for demonstrating the underlying connectivity of MOFs. Table 1, rows 4 and 5, show IRMOF-1 and UiO-67 as illustrative examples of MOFs with their overlaid topologies.

### 3.3 Gas Adsorption Representation

**Figure 3:**
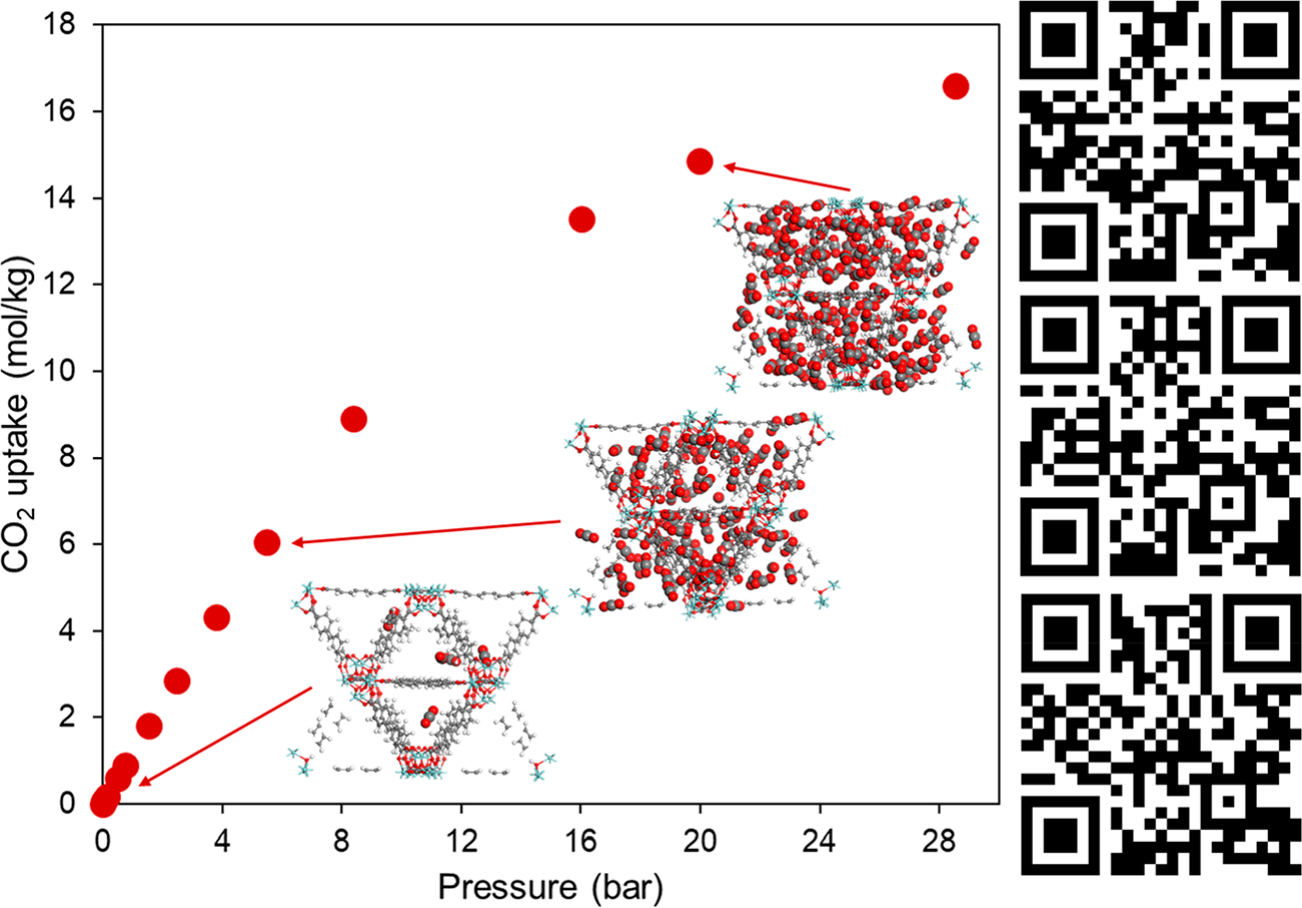
CO_2_ adsorption isotherm simulated in UiO-67 at 298 K. Adsorption snapshots are highlighted at 0.15, 5.5, and 20 bar. QR codes for AR visualization are located adjacent to each snapshot.

To obtain more information from AR modeling, one can run adsorption simulations and generate “snapshots” of gas adsorption sites for visualization in AR at desirable operating temperatures and pressures. Here, we use RASPA^27^ for running Monte Carlo simulations of adsorption in MOFs. Once the simulation at a specific temperature and pressure in the isotherm is equilibrated, RASPA provides CIF and PDB output files for the framework and the adsorbate molecules, and to visualize gas adsorption snapshots, these two files should be merged into a single file followed by the AR development process, as explained in Section 2. Figure 3 demonstrates CO_2_ adsorption isotherm simulated in UiO-67 at room temperature. As can be seen, we can use AR to visualize gas adsorption snapshots as pressure is increased from 0.15 to 20 bar. These snapshots are all produced in RASPA forming the basis of AR model creation for gas adsorption visualization.

In Table 1, rows 6–10, we demonstrate AR representation of CO_2_ and H_2_O adsorption in selected MOFs. By simply scanning the relevant QR code, one can create an immersive experience, investigating how the pore environment and surface chemistry affect the adsorption of CO_2_ or water in MOF-812, CALF-20, UiO-66, HKUST-1, and KAUST-7. In contrast to typical 2D visualization of simulation snapshots, AR can thoroughly capture the clustering of water molecules and the formation of hydrogen bonds, typically seen in hydrophilic MOFs such as in Cu-BTC. In CALF-20, it can be seen that CO_2_ molecules sit tightly within the channels of the oxalic acid linkers where they strongly interact with the metal nodes. In UiO-66, we can use this experience to easily visualize that CO_2_ molecules occupy the tetrahedral cages first at low pressure conditions.

### 3.4 Reception

We demonstrated some of the MOF AR visualizations via QR codes at the first Mediterranean Porous Materials Conference in May 2023 in Crete, Greece, and again at the sixth Annual UK Porous Materials Conference in June 2023 in Sheffield, United Kingdom, receiving approximately 350 views combined. We received many positive comments regarding the quality and clarity of the modeling, and we shared a number of additional QR codes to demonstrate the flexibility of these methods with various structures and gases. We also received constructive feedback for AR demonstration of, e.g., bond vibrations, structural flexibility, and increasing interactivity by implementing measuring tools.

## 4 Conclusion

This article showcases the capability of AR modeling for visualization of MOFs and other porous materials for a variety of applications. We used AR for representing MOFs crystalline structure, their underlying topologies and favorable gas adsorption regions without the need for additional downloads, as the models can be viewed on an Android or iOS smartphone app-free. The technique outlined in this paper and the SI allows anyone to create attractive AR models that can be shared globally by simply distributing a QR code. This freely available, no-cost method is ideal for augmenting MOF posters at conferences, adsorption workshops, and crystal structure presentations as an engaging and interactive experience. Furthermore, the ability to modify the size of AR representations to over 5 m in diameter once placed in a room, establishes the use of AR as an educational tool in the field for furthering understanding of gas adsorption and topological complexities of these intriguing materials. Additional applications of AR include use in research and design for visualization of reactants, intermediates and products in catalysis, crystal engineering and visualization of defects, solvents and irregularities, as well as facilitating collaboration and communication between research groups via the Internet, and even artistic experiences of crystal structure representations.

## Data Availability

All of the structures featured here can be downloaded directly from the Cambridge Structural Database (CSD), which can be obtained from https://www.ccdc.cam.ac.uk/support-and-resources/download-the-csd. RASPA can be freely obtained from https://iraspa.org/raspa/. Blender is available for free at https://www.blender.org/download/. The Jmol freeware can be found at http://jmol.sourceforge.net/download/. Online 3D model hosting platform at https://p3d.in/. These links to the various software packages used are also presented in the SI. We also include a ZIP file of gas adsorption files from the RASPA outputs (where applicable), as well as the FBX files used to create these AR representations.

## Supporting Information Available

Additional guidance for the generation of AR structures. Structure files are also listed below in ZIP files: RASPA output files and FBX files used to create the AR models (PDF)
RASPA output files (ZIP)
FBX files (ZIP)
