# Supplementary material for: Augmented Reality for Enhanced Visualization of MOF Adsorbents
Source: J Chem Inf Model. 2023 Sep 26;63(19):5950–5. doi: 10.1021/acs.jcim.3c01190 (PMC10565814; doi:10.1021/acs.jcim.3c01190)

SUPPORTING INFORMATION

Augmented Reality for Enhanced Visualisation of MOF Adsorbents

Lawson T. Glasby[a], Rama Oktavian[a], Kewei Zhu[b], Joan L. Cordiner[a], Jason C. Cole[c], and Peyman Z. Moghadam[b]*

[a] Department of Chemical and Biological Engineering, The University of Sheffield, Sheffield, S1 3JD, United Kingdom.

[b] Department of Chemical Engineering, University College London, London, WC1E 7JE, United Kingdom.

[c] Cambridge Crystallographic Data Centre, Cambridge, CB2 1EZ, United Kingdom

*Corresponding Author: p.moghadam@ucl.ac.uk

Visualising MOFs with Augmented Reality (AR)

A step-by-step guide to create AR models of MOFs hosted by p3d.in from the Cambridge Structural Database (CSD), with further instructions on how to manipulate RASPA movie files, or topology output files.

Requirements:

CSD Mercury - https://www.ccdc.cam.ac.uk/support-and-resources/download-the-csd/

Jmol - http://jmol.sourceforge.net/download/

Blender - https://www.blender.org/download/

An active p3d.in account – https://p3d.in

(Optional but required for gas adsorption representations)

RASPA - https://iraspa.org/raspa/

(Optional but required for topology representations – choice of either)

CrystalNets - https://github.com/coudertlab/CrystalNets.jl

ToposPro - https://topospro.com/software/topospro/download/

AR File Creation Method

Part A – Selecting and Modifying Files:

1. Install and licence CSD Mercury and ensure the database is the latest version.

2. Select a CSD refcode that you would like to develop an AR interaction for (or have a file available for the structure you are interested in, in PDB, MOL2, or CIF format from any other resource, including RASPA Movie outputs)

3. Open the structure in CSD Mercury by either searching for it by refcode or dragging the file to the viewing window (here we search for BEDYEQ). Ensure that Packing (bottom left) is selected to display the unit cell. This is important as what we see – is what we get.

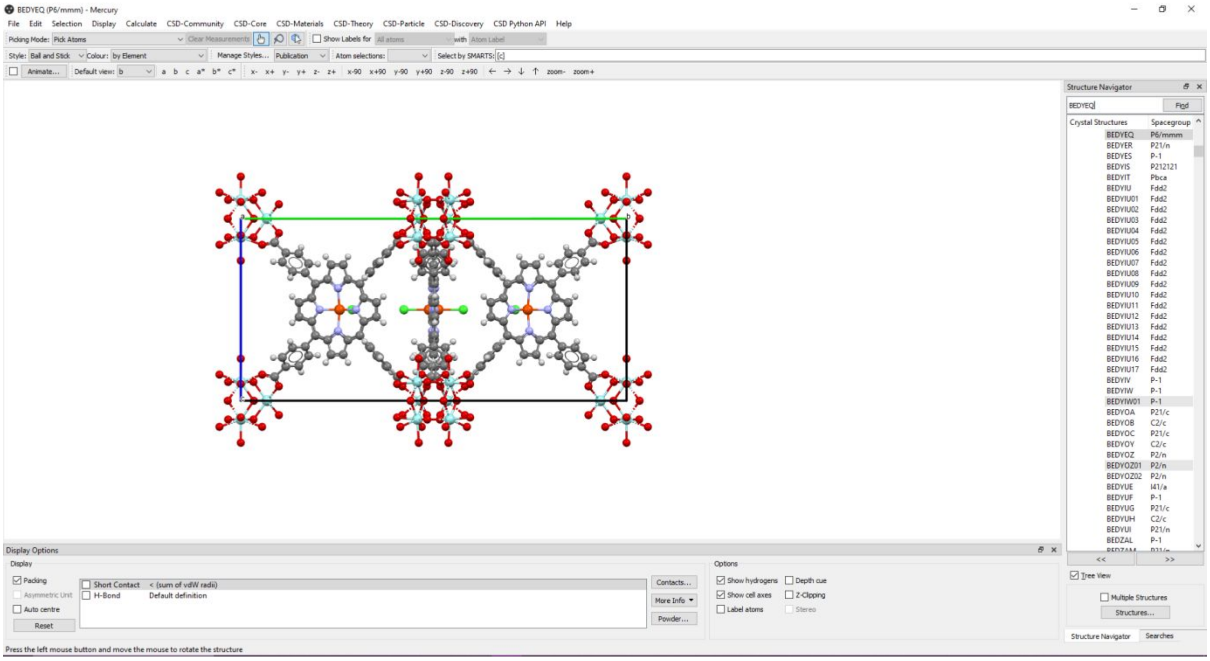

4. Once the file is loaded, make any changes to the structure that you see fit (e.g remove unbound solvents, trim the edges of the unit cell to make it more uniform etc.) You can do this by going to Edit -> Edit Structure. Find Remove at the bottom and click Atoms & Bonds, then click any atom or bond to remove it.

   Note: If you are loading a PDB file, the bonding information may be corrupted. To solve this, go to Edit -> Edit Bond Distance Limit and click Apply. This should reset the bonding, but it is important at this stage to check your structure and manually verify that the bonding is correct. If you find any abnormalities, then they can be corrected using the Edit Structure window as above to remove or change the bond types.

5. When you are content with the structure representation, you should save it as a new PDB file (eg. refcode_new.pdb). Ensure you have made all final structure changes before this step. It is also possible to save the file as a CIF at this stage, it should not make a difference.
6. You can now close CSD Mercury.

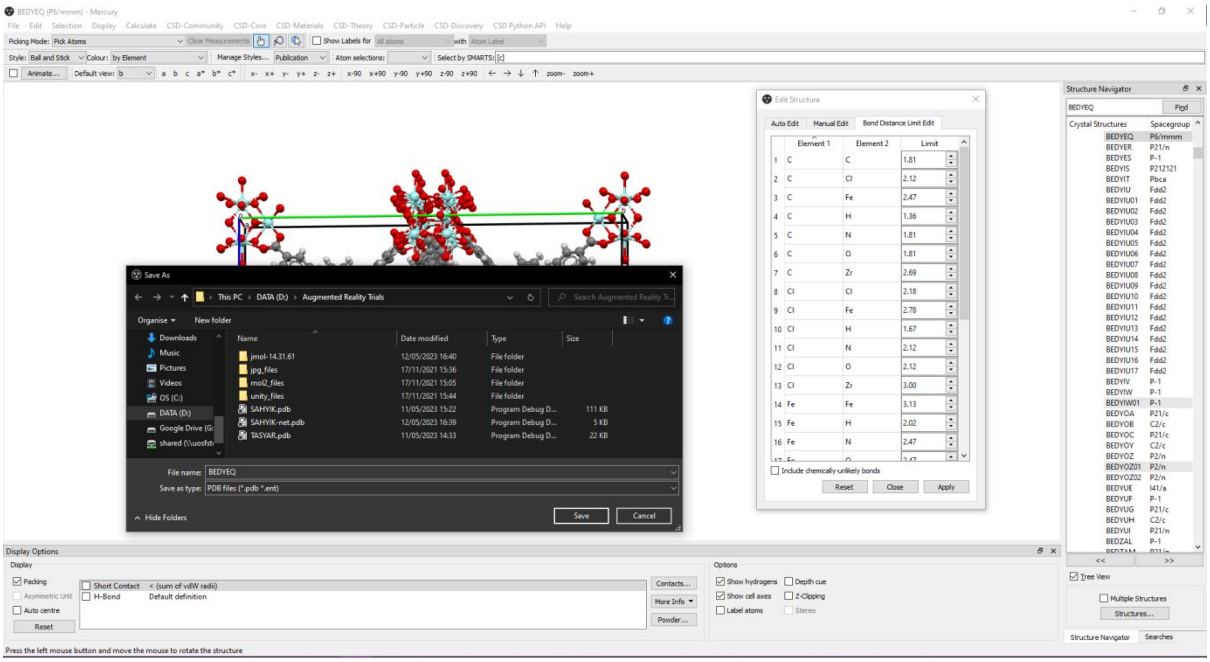

Part B:

1. Open your new PDB file in Jmol.

2. Right Click and go to Style - > Scheme -> Ball and Stick. Make sure the that the representation is in this format as once the file has been saved, it will stay in this representation. (Although you are free to choose whichever format you prefer.)

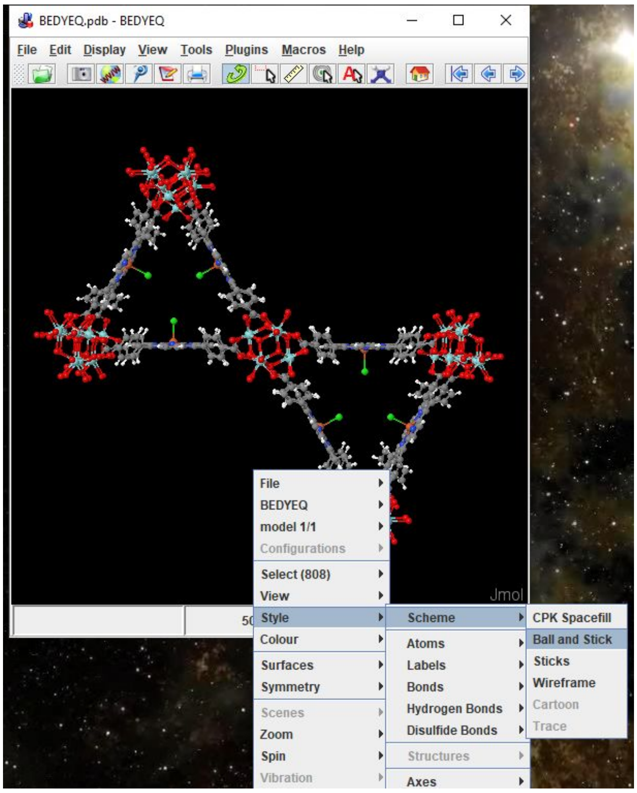

3. Go to File - > Console… This should open the console, where it will load the file name, e.g CSD ENTRY BEDYEQ. Next to the pink $ enter: write new_file_name.obj and press enter.
   This will print an object (OBJ) file into the default Jmol output folder with the name you have assigned it. If the command is incorrect, the text will turn red.

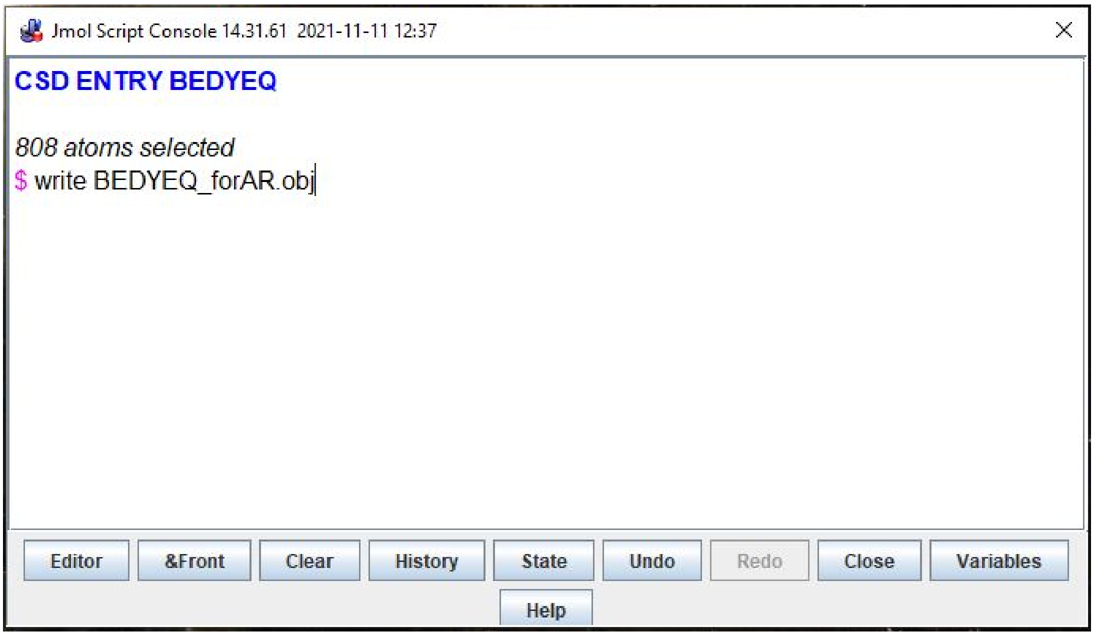

4. Wait for the console to give the OK, and the file path of your new file will be shown on screen.

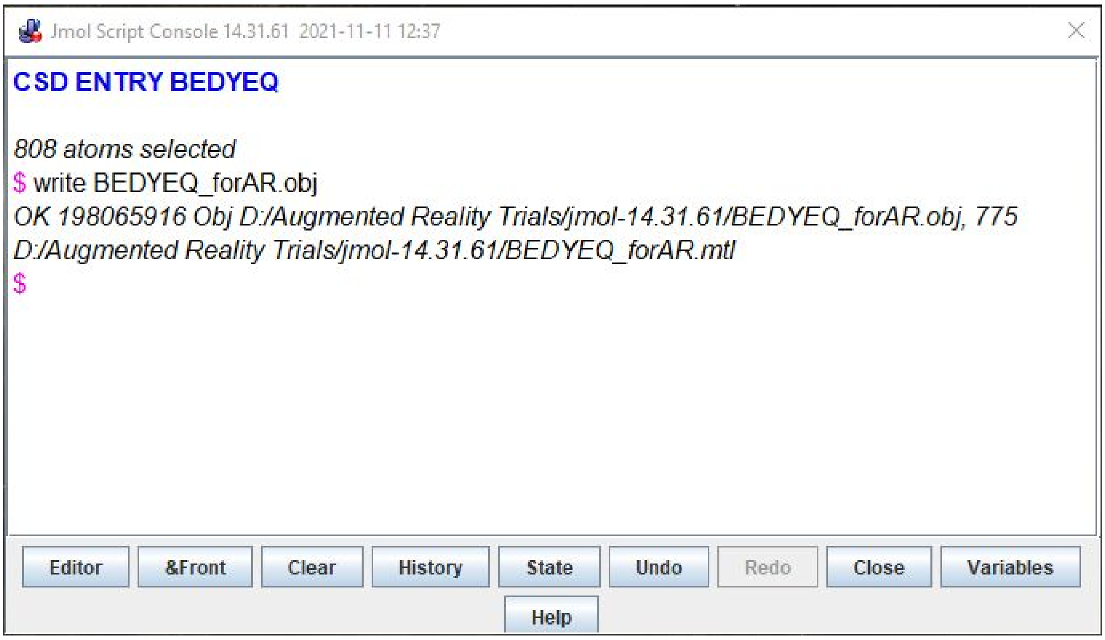

5. You can now close Jmol.

Part C:

1. Open Blender. Select a new general starting platform. Delete the default grey cube by selecting it and pressing delete. In the top right-hand corner, also delete the

camera, and light layers by clicking on them and pressing delete. (This is required to keep file sizes low, so that the detail of the AR figure is kept at a maximum).

2. Go to File - > Import -> Wavefront (.obj) and import your object file from the output folder of Jmol. (You may need to wait for it to render, these files can be >250MB and may take some time to load.)

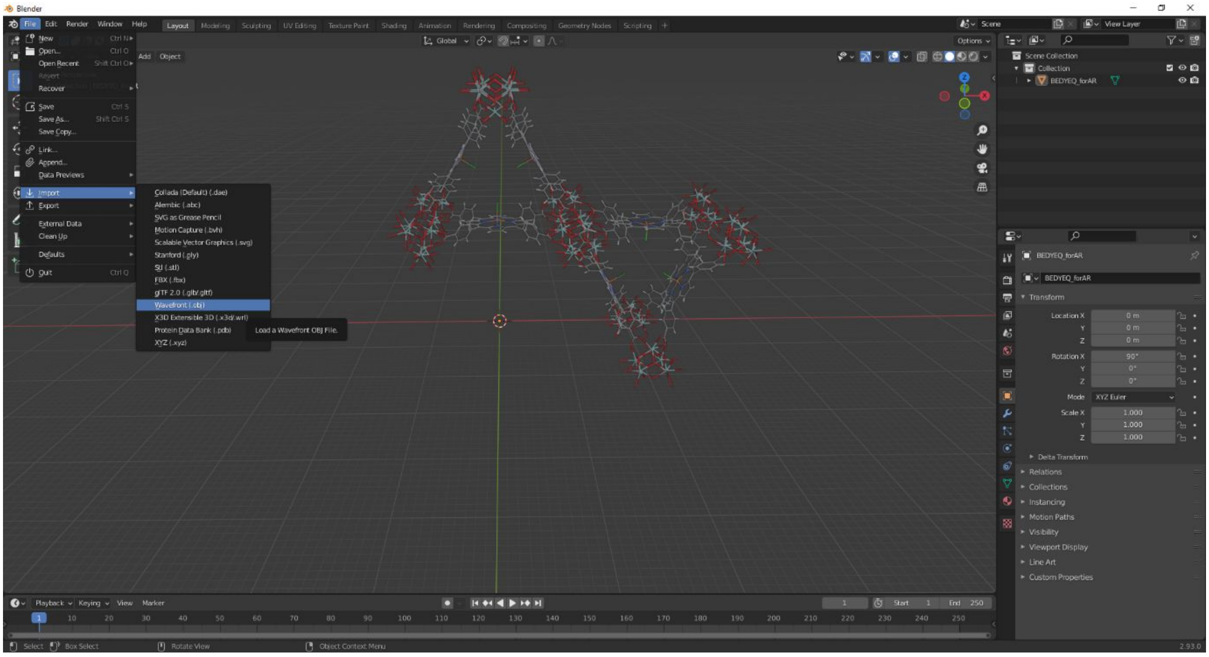

3. Select the object using left click and find the Spanner (Wrench) icon in the right-hand side panel. Here it will ask you to “Add Modifier”.

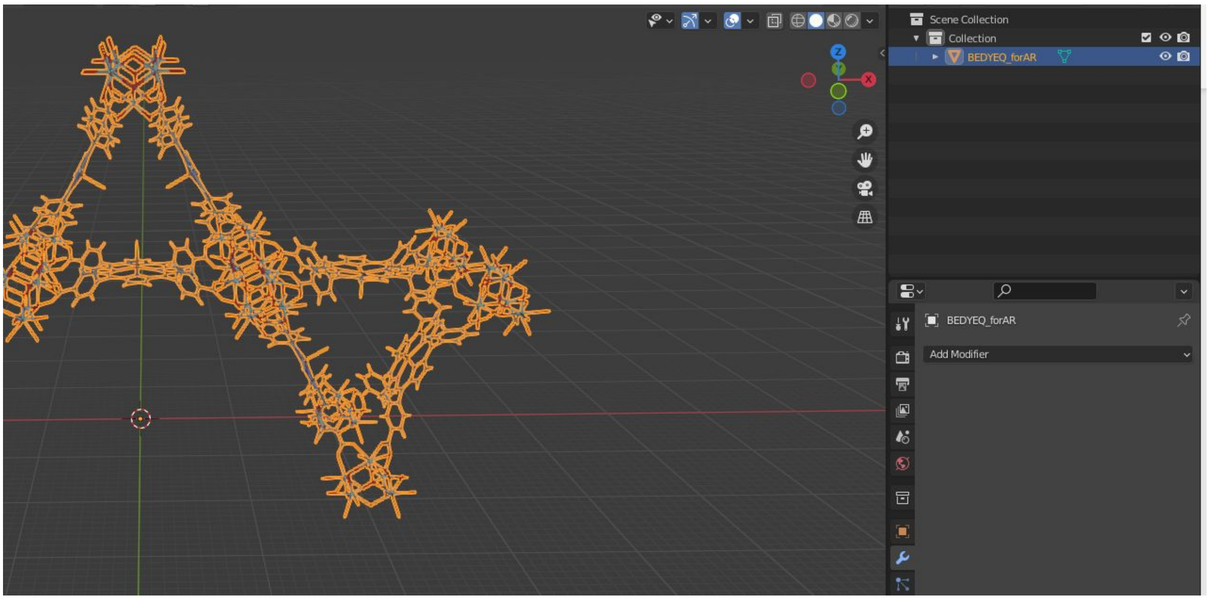

4. Select Add Modifier -> Decimate (in the Generate column). Check the Face Count (FC) of your object – for AR this must be <750,000, if it is higher, calculate the required ratio adjustment to bring it down to an acceptable level.

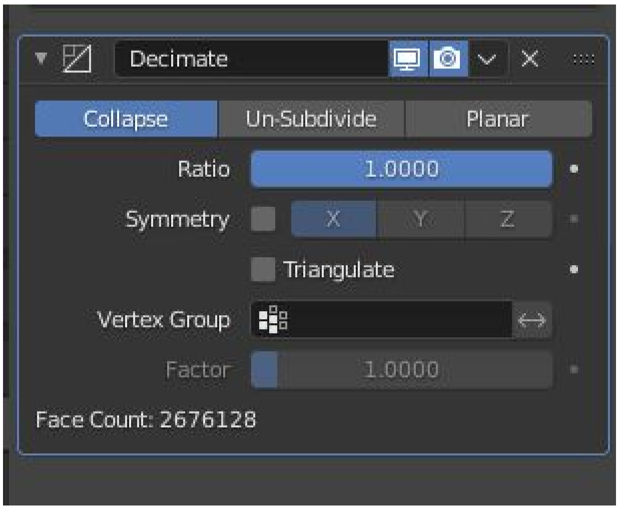

5. In this case, the FC is 3.57 times larger than acceptable. Here we have adjusted the ratio to 0.25 to bring it within an acceptable limit – try to get the FC to as close to 750,000 as possible as to not compromise on the quality of the representation.

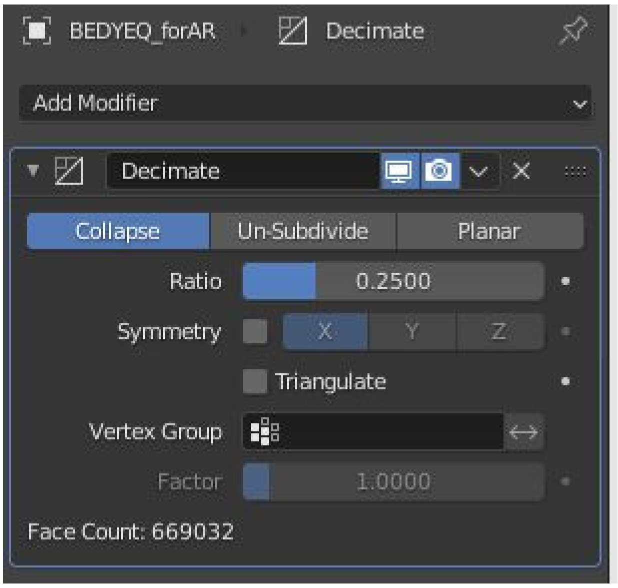

6. Now we need to export the file into a 3D rendering format. Go to File -> Export -> FBX (.fbx) and save the file as something appropriate, e.g refcode_AR.fbx

7. You can now close Blender.

<u>Part D:</u>

1. Go to [p3d.in](p3d.in) and sign up as a Free User. Once you have logged in, you should see the dashboard.

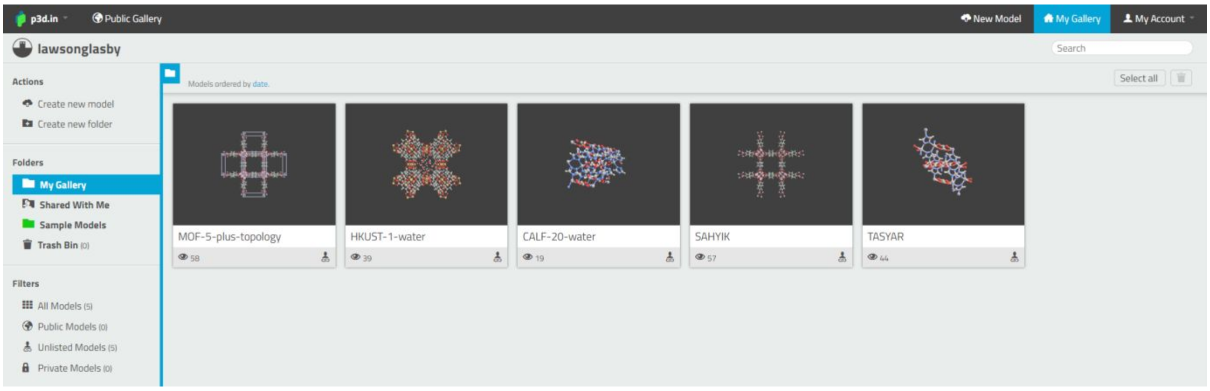

2. From here, select "Create new model" in the top left. Drag your FBX file into the box and wait for it to render. Alternatively, you can upload the file from the file explorer.

   Note: When using the free version your FBX file must be smaller than 50MB. If you use the decimate tool, then it typically will condense the file to somewhere between 7-20MB.

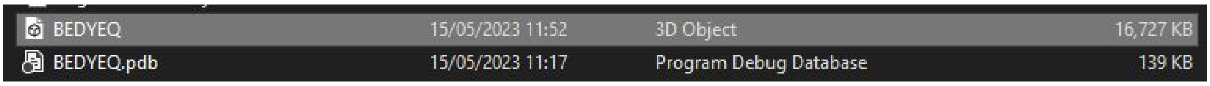

3. Once the model has rendered you can make changes to the colours of the object, and the background by following the settings available to you via the modification pane on the left-hand side of the website.

4. In Mesh and Materials, you will find a list of atoms – the colour of all C atoms will be modified by changing the colour of this entry in the list.

   We feel that changing the atom colours to "Classic" is a better representation than the default "Realistic PBR". These atoms colours can easily be swapped in a drop-down menu.

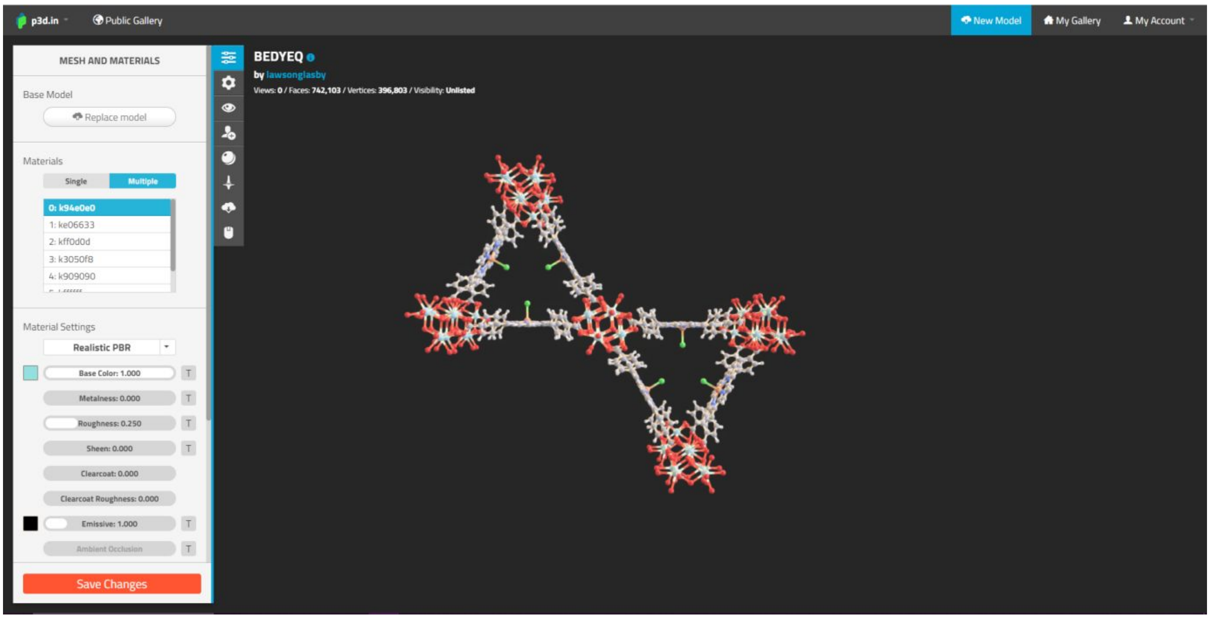

5. Background settings can be changed in Viewer Customisation. Find Viewer Background and set the background colour to white. Ensure in this section that Augmented Reality is set to “Enable”. (This is per your preference but we find either black or white works best, although there are also options to select an environment such as hotel room.)

6. Save changes to exit and publish the structure.

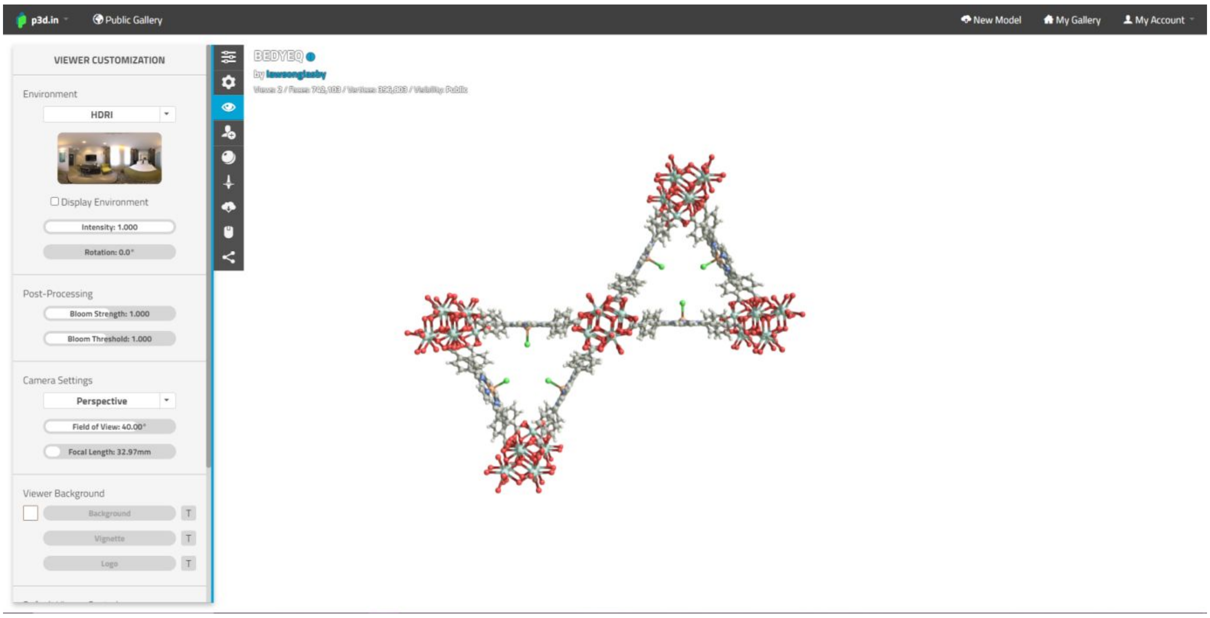

7. Copy the new unique URL from the published structure. You can choose to keep the structure private such that it can only be accessed via that URL (and corresponding QR) or you can publish it publicly, or hide it completely.

<u>Part E:</u>

1. Take the unique URL published from the structure, in the format of p3d.in/xxxxx and visit the-qrcode-generator.com/. Select static QR generator, choose URL and paste the link in the Enter URL box. You can then customise the adjacent QR code.

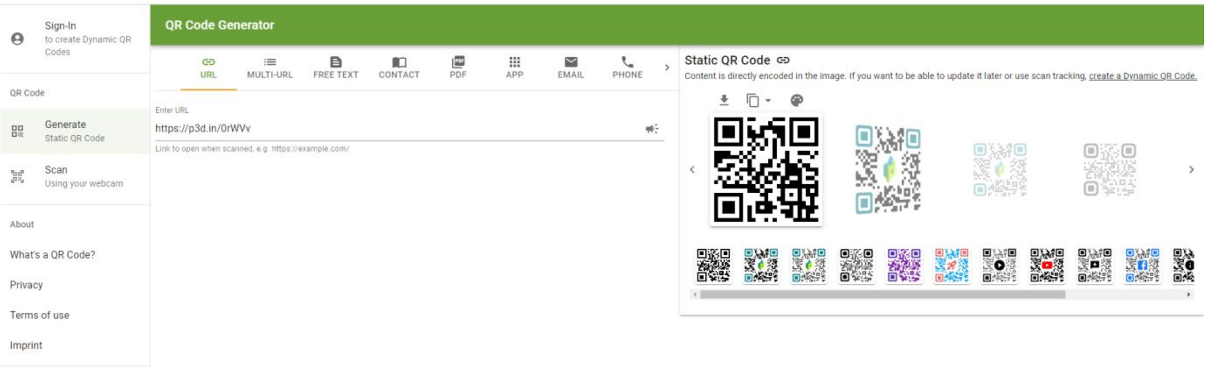

2. Download the 1000px version of the QR code that is unique to your new structure and keep it safe.

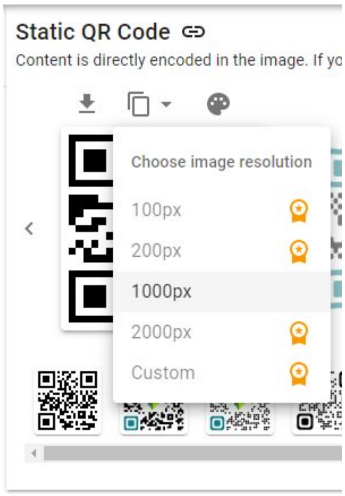

3. Post your QR on research posters, websites, journal articles, and more!

4. If at any time you would like to modify your structure, you can edit the file corresponding to this QR code. Log back in to p3d.in and edit the relevant link, here you can upload new FBX files entirely and retain the same URL, you can also modify any of the settings at any time and they will be applied as soon as you save the entry again.

RASPA AR file generation for gas adsorption visualisation

1. Download RASPA. (If you are not familiar with RASPA there are several online guides and workshops available on the iRASPA website linked at the beginning of this document).

2. Submit a CIF to RASPA and define your input parameters. Ensure that the “Movie” parameter is set to “yes” and specify the number of iterations between snapshots.

```
SimulationType MonteCarlo
NumberOfCycles 20000
NumberOfInitializationCycles 20000
PrintEvery 5000
Restartfile no

RemoveAtomNumberCodeFromLabel yes

Movies yes
WriteMoviesEvery 1000
ComputeRDF yes
WriteRDFEvery 1000
RDFHistogramSize 100
RDFRange 12.0

CutOffVDW 12.8
Forcefield MOF_DFF_UFF_rama

Framework 0
FrameworkName SIFSIX_unopt_charge
ChargeMethod Ewald
UseChargesFromCIFFile yes

UnitCells 4 4 4
HeliumVoidFraction 0.297
ExternalTemperature 298
ExternalPressure 100000

Component 0 MoleculeName             CO2
            MoleculeDefinition       TraPPE
            IdealGasRosenbluthWeight 1.0
            TranslationProbability   1.0
            RotationProbability      1.0
            ReinsertionProbability   1.o
            SwapProbability          1.0
            CreateNumberOfMolecules  0
```

3. Run RASPA. Once the simulations are completed, a Movie output folder will have been created containing the snapshots at each point of the isotherm. Select the one you would like to represent in AR and copy either the CIF or PDB file to a new folder where you will begin to create the AR representations.

4. Follow the steps in this guide from Part A.

CrystalNets AR file generation to visualise topology

Part I: Obtaining OBJ format topological nets.

1. Download the relevant CIF file for your chosen structure.

2. Visit https://progs.coudert.name/topology and upload the CIF, cycle through the main options and select settings that are relevant to the chosen crystal. For a MOF, we selected the following settings:

   Structure Type: MOF, Bonding: Auto, Clusterings: SingleNodes, Exports: Trimmed, Subnets, Clusters

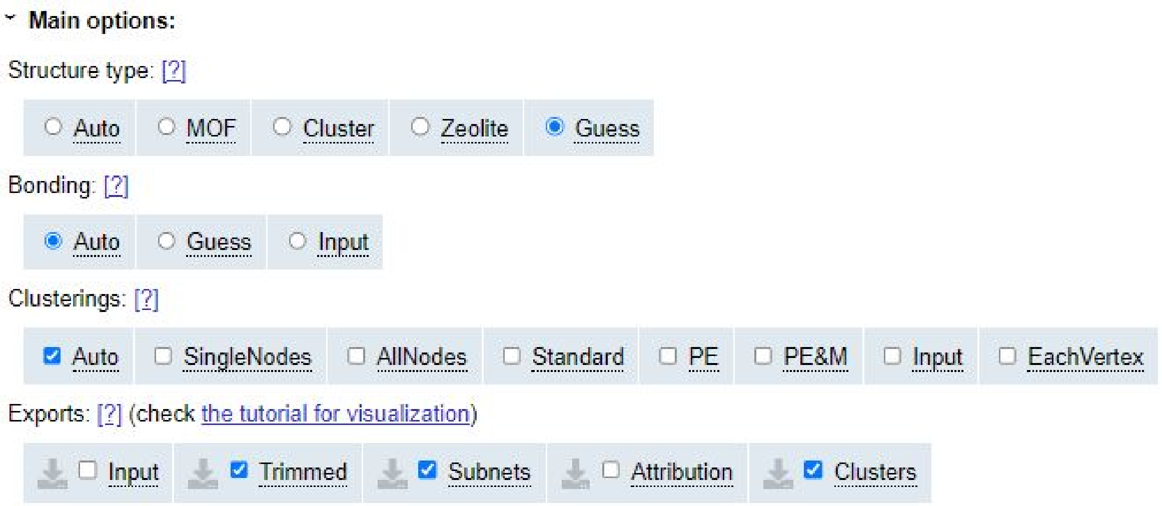

3. The output file will be obtained in a VTF format and can be downloaded from the subnets section.
   Note: To view these nets in Mercury, it is then necessary to convert the output VTF file into a MOL2 format. VTF files cannot be opened in Merucry or Jmol, however MOL2 can be opened in Jmol and converted into an OBJ file, as in Part B of this guide.

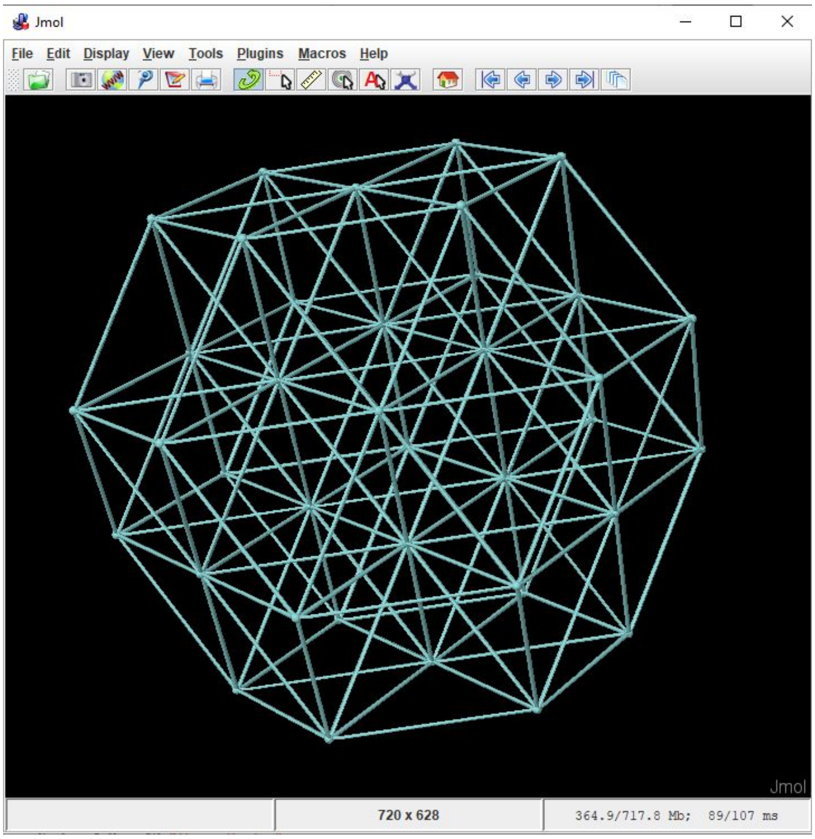

4. Open your newly created MOL2 file in Jmol and follow the steps in Part B using the instructions for the console in Jmol to obtain an OBJ file from the MOL2 input.

Part II: Combining OBJ nets and OBJ crystals in Blender.

1. Once the OBJ file of the net has been created, it is necessary to create the OBJ of the crystal structure itself, although these stages can be completed in either order. For the AR representation of the original crystal, follow Parts A and B of this guide and return here to combine the two files.

2. Begin by importing the crystal OBJ file. Go to File - > Import -> Wavefront (.obj) and import your crystal object file from the output folder of Jmol. (You may need to wait for it to render, these files can be >250MB and may take some time to load.)

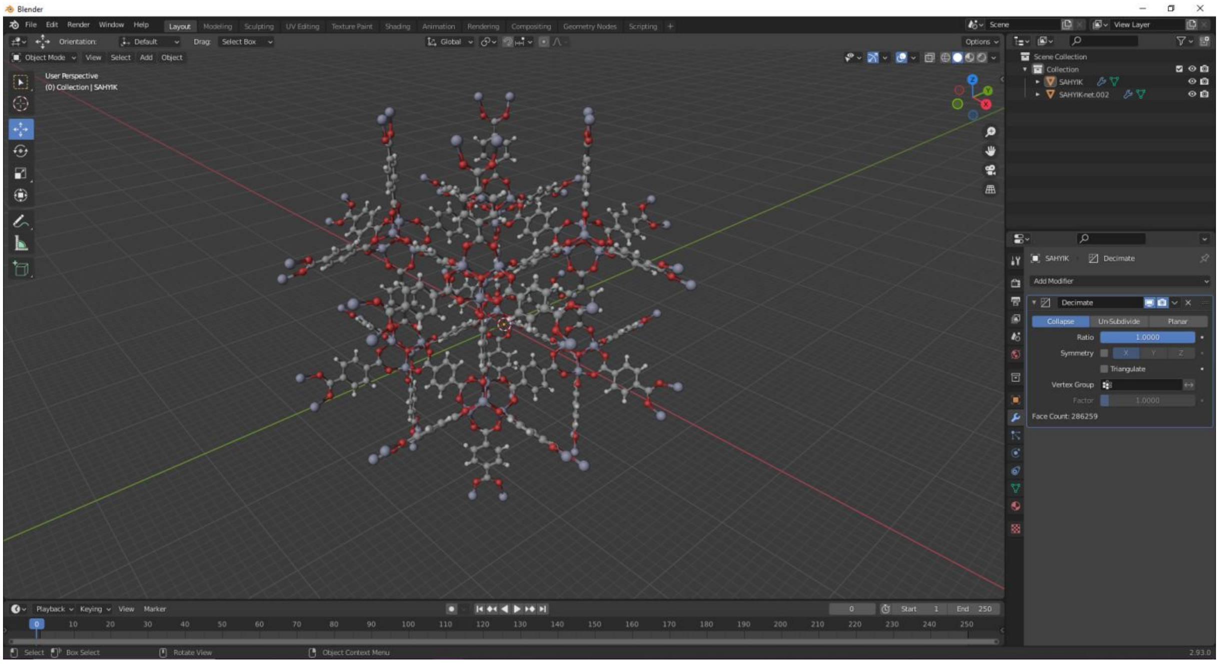

3. Now the OBJ files can be combined in Blender if the same original CIF was used to create both files. Some modification may be required in Blender to ensure the underlying net is configured in the correct position, although if the exact same CIFs are used this is unlikely. Import the nets OBJ file. Go to File - > Import -> Wavefront (.obj) and import your topology net object file.

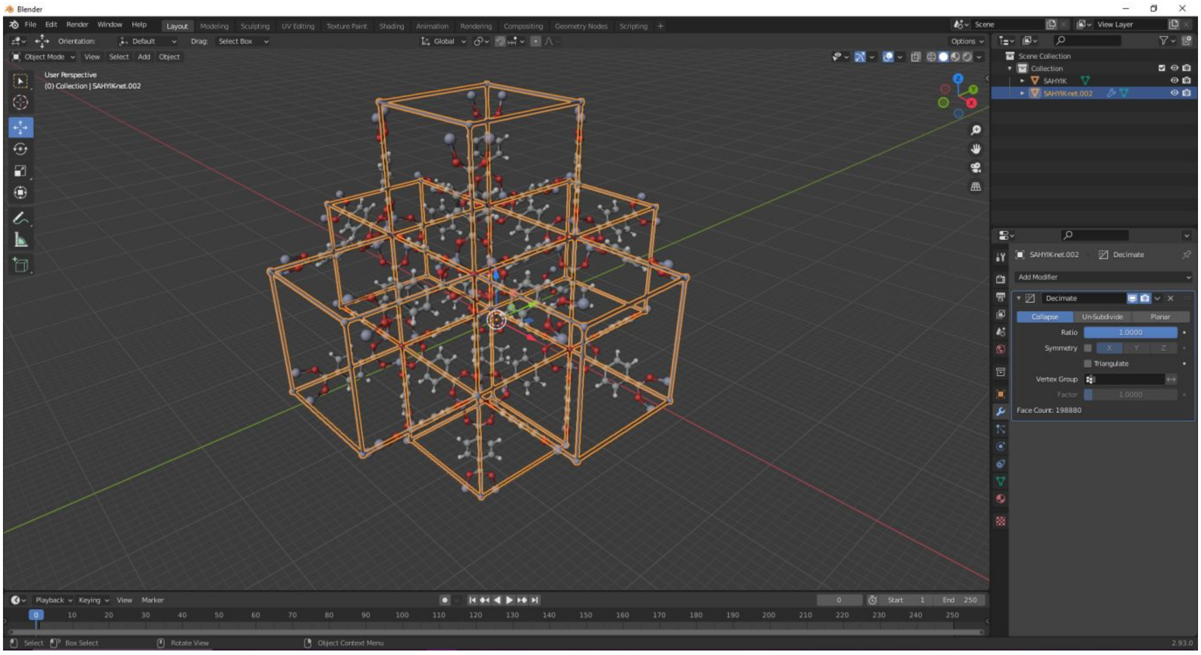

Note: These stages draw many similarities with Part C, with a few additional steps. If the net requires some adjustment, it can easily be moved around and re-scaled in Blender.

4. Once the initial structures have been imported, to ensure that the combined files will render in Augmented Reality we need to add the total face counts for both OBJ files together and decimate them both so that the sum of faces does not exceed 750,000.

   Select the object using left click and find the Spanner (Wrench) icon in the right-hand side panel. Here it will ask you to "Add Modifier". Select Add Modifier -> Decimate (in the Generate column). Calculate the required ratio adjustment to bring it down to an acceptable level.

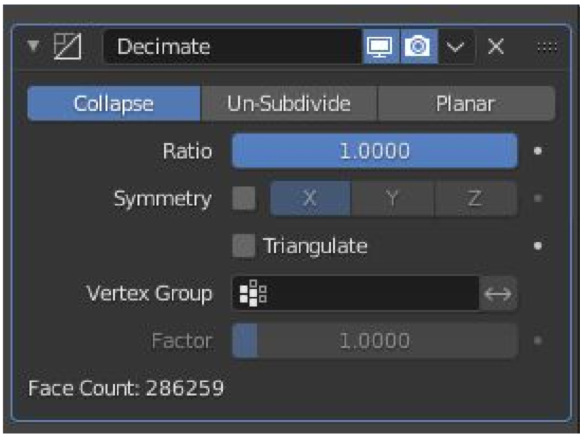

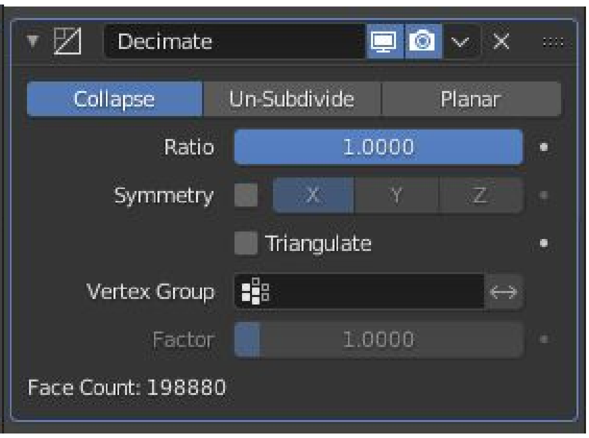

Note: We would recommend decimating the underlying net more than the crystal structure as the quality of the render for the net is less significant. Ensure that the SUM of face counts does not exceed 750,000!

5. Once the face count is at an acceptable level, the combined structures can be exported as an FBX file. Go to File -> Export -> FBX (.fbx) and save the file as something appropriate, e.g refcode_topology_AR.fbx

6. Close Blender and follow Part E to complete the upload to p3d for AR visualisation.

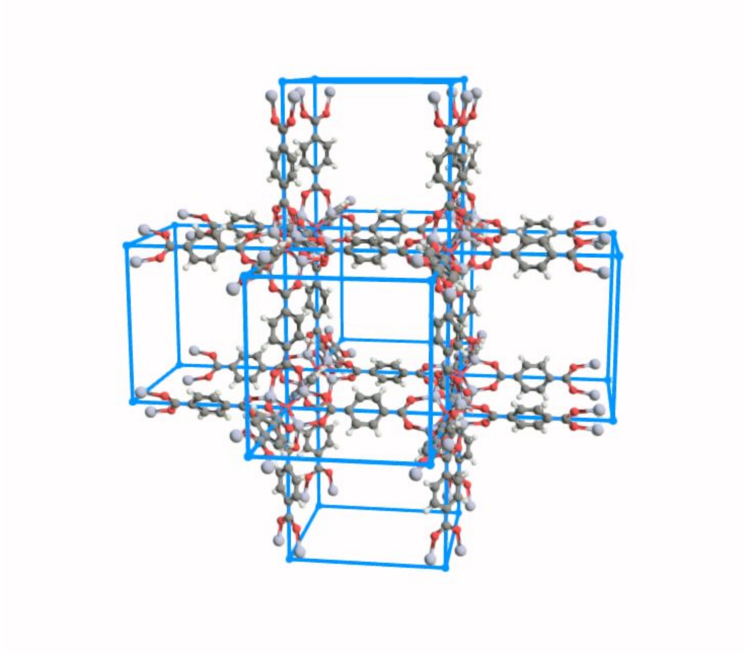

Supplement: Supplementary file 1 — ci3c01190_si_001.pdf [file ci3c01190_si_001.pdf]
